# Electrophysiological and Histological Characterization of Rod-Cone Retinal Degeneration and Microglia Activation in a Mouse Model of Mucopolysaccharidosis Type IIIB

**DOI:** 10.1038/srep17143

**Published:** 2015-11-26

**Authors:** Dennis Y. Tse, Parisa Lotfi, David L. Simons, Marco Sardiello, Samuel M. Wu

**Affiliations:** 1 Department of Ophthalmology, Cullen Eye Institute, Baylor College of Medicine, Houston TX, USA; 2 Department of Human and Molecular Genetics, Baylor College of Medicine, Jan and Dun Duncan Neurological Research Institute, Texas Children’s Hospital, Houston TX, USA; 3 School of Optometry, The Hong Kong Polytechnic University, Hong Kong

## Abstract

Sanfilippo syndrome Type B or Mucopolysaccharidosis IIIB (MPS IIIB) is a neurodegenerative autosomal recessive lysosomal storage disorder in which patients suffer severe vision loss from associated retinopathy. Here we sought to study the underlying retinal functional and morphological changes associated with MPS IIIB disease progression using the established model of MPS IIIB, the B6.129S6-Naglu(tm1Efn)/J mouse line. Electroretinogram (ERG) was recorded from MPS IIIB and wild-type (WT) mice at the age of 28 and 46 weeks, and retinal tissues were subsequently collected for immunohistochemistry analysis. At the 28th week, rod a- and b-wave amplitudes were significantly diminished in MPS IIIB compared to WT mice. The cone a- and b-waves of MPS IIIB mice were not significantly different from those of the control at the 28th week but were significantly diminished at the 46th week, when MPS IIIB mice showed a major loss of rods and rod bipolar cells in both central and peripheral regions and a minor loss of cones in the periphery. Activation of microglia and neovascularization were also detected in the MPS IIIB retina. The new findings that cones and rod bipolar cells also undergo degeneration, and that retinal microglia are activated, will inform future development of therapeutic strategies.

Mucopolysaccharidoses (MPS) are part of the lysosomal storage disease family, a large group of more than 50 clinically recognized conditions resulting from genetic defects of metabolism affecting lysosomes^1^,^2^. The lysosome acts as a highly coordinated recycling center that processes metabolic waste into substances that the cell can utilize via specialized enzymes^3^,^4^. Patients with MPS have mutations in one of the genes encoding lysosomal enzymes needed to break down glycosaminoglycans (GAGs), long unbranched polysaccharides consisting of a repeating disaccharide unit. In MPS Type III (MPS III), or Sanfilippo syndrome, one of the four enzymes needed to degrade the GAG heparan sulfate (HS) is deficient.

MPS III diseases are pathophysiologically characterized by the accumulation of undegraded HS in lysosomes and the excretion of its derived polysaccharide fragments in body fluids. Heparan sulfate, a component of proteoglycans, is ubiquitously distributed in the cell membrane and extracellular matrix. Its water-binding property plays an important role in tissue structure. It also play roles in various physiologic and pathophysiologic processes including cell growth and development, infection, cell-cell interaction, lipid metabolism and coagulation by interacting with proteins such as growth factors, chemokines and proteases^5^. The central nervous system (CNS) is the main organ affected in MPS III, though the exact mechanism by which accumulation of HS leads to CNS degeneration remains elusive. In addition to the accumulation of HS, secondary accumulation of gangliosides has also been suggested to contribute to CNS damage^6^,^7^. The inflammatory response of microglial cells in response to accumulated gangliosides has been suggested to exacerbate neuronal damage^8^,^9^.

MPS III is the most frequently occurring MPS type and is inherited in an autosomal recessive manner. Birth incidence varies geographically, with reported rates ranging from 0.28 to 4.1 per 100,000 birth^10^. MPS III may be further classified into subtypes A–D according to the identity of the defective enzyme. The incidence of all four MPS III sub-types combined is estimated to be 1 in 70,000 births. There is currently no cure for this vision-threatening and fatal disease. Type B is most prevalent in south-east Europe, while type A is most common in north-west Europe^11^,^12^,^13^. Types C and D are relatively rare. Possibly complicated by the wide spectrum of clinical phenotypes, disease severity and numerous polymorphisms^14^,^15^, the four MPS III subtypes display extensive overlaps from a clinical standpoint^16^, except that type A has earlier onset, faster progression and shorter lifespan^17^.

The first signs and symptoms of MPS III usually present as developmental delay, behavior abnormality and sleep disturbance between the age of 1 and 6^18^,^19^. Patients often show progressive developmental regression in speech, mental/cognitive and motor functions and eventually become bedridden. Visual impairment is common in most types of MPS, including MPS III^20^. In contrast to MPS I, changes in the anterior eye usually do not impair vision in MPS III^21^. Cornea and lens opacification are usually subclinical in MPS III, and intraocular pressure is typically normal. The most prominent features are pigmentary retinal degeneration and associated electroretinogram changes. Histopathology showed marked photoreceptor loss without observable lesion in ganglion cell and optic nerve in older patients, resembling retinitis pigmentosa^22^,^23^,^24^,^25^. Unfortunately, the natural time course of visual impairment for MPS III is largely unknown because visual testing is extremely difficult in young patients due to behavior problems and cognitive impairment. Available data are limited and are usually in the form of isolated case studies or series.

An animal model of MPS III was first reported in mice by disrupting the α-N-acetylglucosaminidase (*Naglu*) gene^26^, which is mutated in MPS IIIB patients. Such mouse model recapitulated the human disease by demonstrating accumulation of abnormal lysosomal inclusions, HS, and gangliosides in multiple organs as well as reduced lifespan and increased anxiety response^8^,^27^. Heldermon *et al*.^28^ phenotyped the general sensory and motor functions of the model in 2007 in a study which included a screening of electroretinogram (ERG) and basic retinal histology. The mice were found to have a suppressed rod b-wave when measured with a strong rod-saturating flash. There was progressive loss of photoreceptor cells in the outer retina and aberrant lysosomal storage in some non-neuronal cells in the inner retina. No studies, however, have analyzed the photoreceptor originated a-wave or performed cell-specific and quantitative histology analysis yet.

In the present study, to fill in the above gaps of knowledge we examined the ocular functional changes in depth using a comprehensive ERG protocol covering scotopic, mesopic and photopic intensities. The a-wave and b-wave were mathematically modeled to provide a more accurate account of the underlying functional changes in photoreceptors and bipolar cells. We also sought to study the cell-specific changes in histology by using immunohistochemistry (IHC). ERG a-wave and b-wave analysis showed that the rod pathway was affected earlier than the cone pathway in the MPS IIIB mice. IHC revealed massive generalized loss of rods, and a minor localized loss of cones at a late stage of the disease (46^th^ week). The data also revealed an unexpected generalized loss of rod bipolar cells, and the activation of retinal microglia. This report provides quantitative data on the natural time course of retinal degeneration in the MPS IIIB mouse model, and is the first to show the involvement of microglia activation in the degeneration.

The retina is an extension of the central nervous system^29^. The ERG is a direct functional measurement of the retinal neurons which has the advantages of being non-invasive, repeatable and quantitative, and would be particularly instrumental to evaluate the effectiveness of various emerging therapeutic options for this neurodegenerative disease (see Mauri *et al*. for a review^30^).

## Methods

### Animal model and handling

Mucopolysaccharidosis type IIIB (MPS IIIB; Sanfilippo syndrome type B) mice (B6.129S6- *Naglu*^tm1Efn/J^) and their age-matched wild-type cohort were purchased from the Jackson lab. All experimental protocols related to animal work were approved by the Institutional Animal Care and Use Committee (IACUC) of Baylor College of Medicine (Protocol AN-1979). All methods were carried out in accordance with the approved welfare guideline of the IACUC.

### Electroretinogram (ERG) recordings

Scotopic ERGs were recorded bilaterally from MPS IIIB mice (n = 8) and their wild-type (n = 7) littermates at ages 28 and 46 weeks. Prior to testing, mice were allowed to dark-adapt overnight. Under dim red light, mice were anaesthetized with a weight-based intraperitoneal injection of solution containing ketamine (46 mg/ml), xylazine (9.2 mg/ml), and acepromazine (0.77 mg/ml). Both pupils were dilated with a drop each of 1% tropicamide and 2.5% phenylephrine and the corneas anesthetized with 0.5% proparacaine hydrochloride. Mice were placed inside a Ganzfeld dome coated with highly reflective white paint (Munsell Paint, New Windsor, NY) on a heating pad maintained at 39 °C. A drop of 2.5% methylcellulose gel (Goniosoft, Ocusoft Inc, TX) was applied to the eye and a blunt platinum rod electrode placed in contact with the center of each cornea. Ground and reference platinum subdermal electrodes were gently inserted in the tail and the forehead, respectively. The mice were then allowed to remain in complete darkness for 5 minutes prior to initiating the experiment.

Half millisecond square flashes for scotopic a-wave and b-wave measurements were produced by cyan light emitting diodes of 503 nm peak wavelength, calibrated with a photometer (ILT1700 International Light, MA) and converted to the unit photoisomerizations/rod (R*/rod)^31^,^32^, where 1 scot cd m^2 = 581 photoisomerizations/rod/s. At the lowest intensity, 25 responses were averaged with a delay of 2 seconds between each flash. As the intensity of the flash increased, fewer responses were averaged with a longer delay between flashes. At the end of the scotopic protocol, a pair of 1500W xenon lamps (Novatron, Dallas, TX) attenuated with apertures and diffusers were used to produce two saturating light stimuli. Since rods are more sensitive than cones, the rod ERG was measured by using single flash stimuli with a strength below the operative range of cones, while the cone ERG was measured by a paired-flash protocol using the xenon flashes^33^. In the paired flashes protocol, an initial conditioning flash (4.6 × 10^6^ R* per M-cone) saturates both rods and cones 2 sec before a probe flash. The ERG recorded by the probe flash (1.8 × 10^6^ R* per M-cone) is attributed to responses driven by the cones because cones recover faster than rods.

Electrical signals were amplified with a Grass P122 amplifier (Grass Instruments, West Warwick, RI) and band-pass filtered from 0.1 to 1,000 Hz. Data was digitized with a National Instruments data acquisition unit (USB-6216, National Instruments, TX) at a 10 kHz sampling rate, and analyzed with custom Matlab code (MathWorks, Natick, MA). To remove oscillatory potentials before fitting, the scotopic b-wave was digitally filtered using the *filtfilt* function in Matlab (low-pass filter; Fc = 60 Hz). The a-wave was measured from baseline to trough of the initial negative deflection (unfiltered) and the b-wave was measured from the a-wave trough to the peak of the subsequent positive deflection (filtered). Stimulus strengths are displayed in log units where 1E + 0 = 1 photoisomerization/rod. Two tails statistical analyses were performed using unpaired t-test or Welch’s t-test in SPSS Version 20 (IBM). Alpha level is set at 0.05.

### Retinal Immunohistochemistry (IHC)

Eyes were enucleated under deep anesthesia, then mice were immediately euthanized by an overdose of anesthesia. The eyes were marked for orientations and carefully dissected to isolate whole retina, which was then incubated in 4% paraformaldehyde (Electron Microscopy Science, Fort Washington, PA) in phosphate buffer (DPBS, pH7.4, Invitrogen, La Jolla, CA) at room temperature for 45 minutes for fixation.

**Figure 1:**
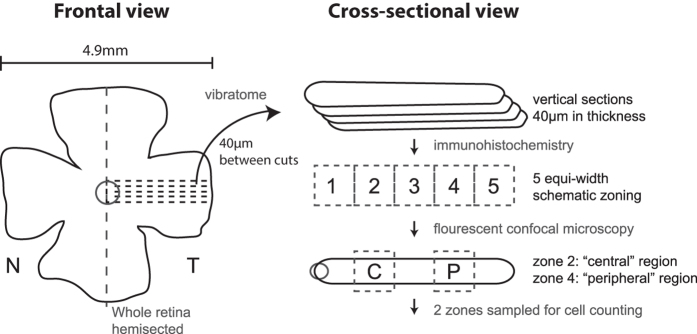
(**a**) Workflow for immunohistochemistry and cell counting. Whole retina was carefully isolated and hemisected. The temporal half was sectioned using vibrating microtome into vertical sections. The distance between each section was set at 40 μm. Dotted lines denote the schematic locations of the cuts. Sections containing the optic nerve head was processed with antibodies and florescent staining, and then mounted on slides. Being zoned into five zones of equal width, the second and the fourth zones from the optic nerve were respectively designated as the “central region” and “peripheral region” and were photographed by a confocal microscope for cell counting.

For the quantification of activated microglia in the inflammation assay, whole-mount retina was tile-scanned with a 10× objective. Further to quantify the percentage occupancy of two layers of Iba-1 positive cells separately, four regions of each flat retina were imaged using 32 um thick z-stack with 1 um interval (33 slices) with a 40× objective. Then images were quantified using imageJ (33 slices/region; 4 region/retina; 2 retina/animal and 3 animals/genotype). Average occupying Iba-1 area (%) was separately calculated in each region (GCL-IPL and OPL). For the cell profiling study, whole retina was further processed into retinal vertical sections. To control for variability from regional retinal difference, the dissection and handling procedures were designed to ensure that the same regions on the retina were being assessed consistently among samples (Fig. 1). The whole retina was first hemisected, then the temporal half of the retina was cut using a microtome (Vibratome; Leica Microsystems, Bannockburn, IL) into 40 μm vertical sections. The samples were then blocked with 10% donkey serum (Jackson Immunoresearch, West Grove, PA) in TBS (DPBS with 0.5% Triton X-100 (Sigma) and 0.1% sodium azide (Sigma), pH 7.2) at 4 °C overnight to reduce nonspecific labeling.

An indirect antibody method was used for the IHC. The whole-mount retina or free-floating sections were incubated with primary antibodies for 4 days at 4 °C in TBS in the presence of 3% donkey serum. Controls lacking primary antibodies were processed as well. After rinsing several times, the samples were transferred and incubated in 3% normal donkey serum-TBS solution containing donkey-host secondary antibodies conjugated with Cy3 (1:200, Jackson Immunoresearch) or Alexa Fluor 488 (1:200, Molecular Probes, Eugene, OR) at 4 °C overnight. A fluorescent nuclear dye, TO-PRO3 (Molecular Probe, Eugene, OR, Cat. No. T3605; dilution 1:3000)^34^, was used to stain the nuclei in the. Rod bipolar cells were immuno-labeled with mouse antibody against PKCα (BD Transduction Lab, San Jose, CA, Cat. No. 610107; dilution 1:250)^35^. Cone cell bodies were immuno-labeled with rabbit antibody against GNAT2 (Santa Cruz Biotechnology, Santa Cruz, CA, Cat. No. SC-390; dilution 1:100)^36^,^37^. Activated microglial cells were immune-labeled with rabbit antibody against Iba-1 (Wako Chemicals, Richmond, VA, Cat. No. 019-19741; dilution 1:500)^38^,^39^. Astrocytes and Müller cells end-feet were immune-labeled with chicken anti-GFAP (Millipore, MA, Cat. No. AB5541; dilution 1:500)^39^. Alexa-488 conjugated Isolectin B4 from Griffonia simpliciflia (GS-IB4) was used to stain the retinal vasculature (Invitrogen, Grand Island, NY, Cat. No. I121411; dilution 1:200)^40^. After rinsing several times, the samples were mounted with Vectashield medium (Vector Laboratories, Burlingame, CA), coverslipped and analyzed with confocal laser scanning microscopes (LSM 510/LSM700; Zeiss, Thornwood, NY). Images of the vertical sections were acquired with Zeiss LSM software using 40× and 63× oil-immersion objectives. Two designated regions on the sections, representing central and peripheral retina, were photographed. Adobe Photoshop CS5 (Adobe Systems, San Jose, CA) was used to apply uniform brightness and contrast adjustments and to crop images. To measure the number of cone photoreceptors (GNAT2 positive cells) and rod bipolar cells (PKCα positive cells), cells with positive staining were counted in a 200 μm segment, using the ruler and cell counter in ImageJ. The dimensions of the outer nuclear layer and the inner nuclear layer were also measured using ImageJ. Images of the whole mounted retina (Iba-1 staining) were acquired using the tile-scan feature of the Zeiss Zen software with a 10× objective. Iba-1-positive cell density and percentage area were analyzed using ImageJ.

## Results

### Electroretinography (ERG)

#### Function of the photoreceptors─a-wave

**Figure 2:**
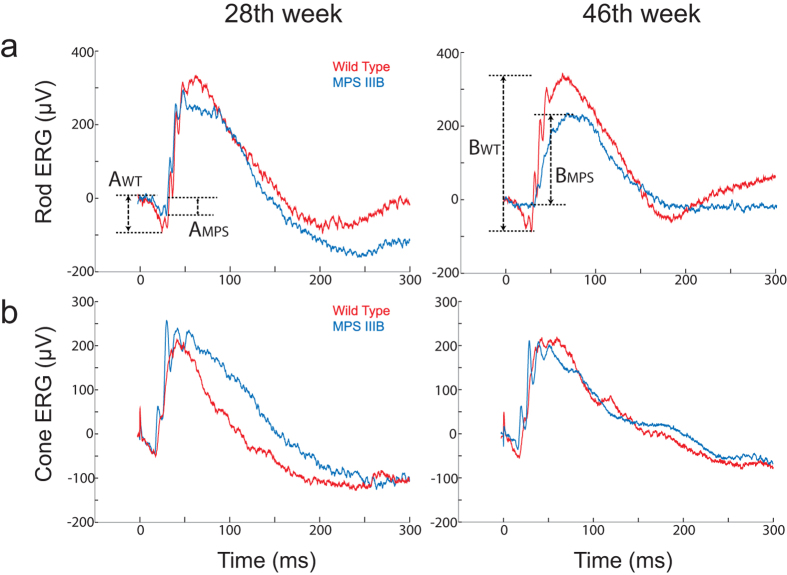
Representative raw ERG traces recorded from wild-type and MPS IIIB mice at the 28^th^ and the 46^th^ week showing the definitions for a- and b-waves. (**a**) Isolated rod ERG measured with a stimulus below the operative range of cones. Amplitudes of the a-waves of wild-type and MPSIII animals are designated as A_WT_ and A_MPS_, respectively. (**b**) Isolated cone ERG measured using a paired flash protocol in which rods were saturated by the conditioning flash. Amplitudes of the b-waves of wild-type and MPSIII animals are designated as B_WT_ and B_MPS_, respectively.

**Figure 3:**
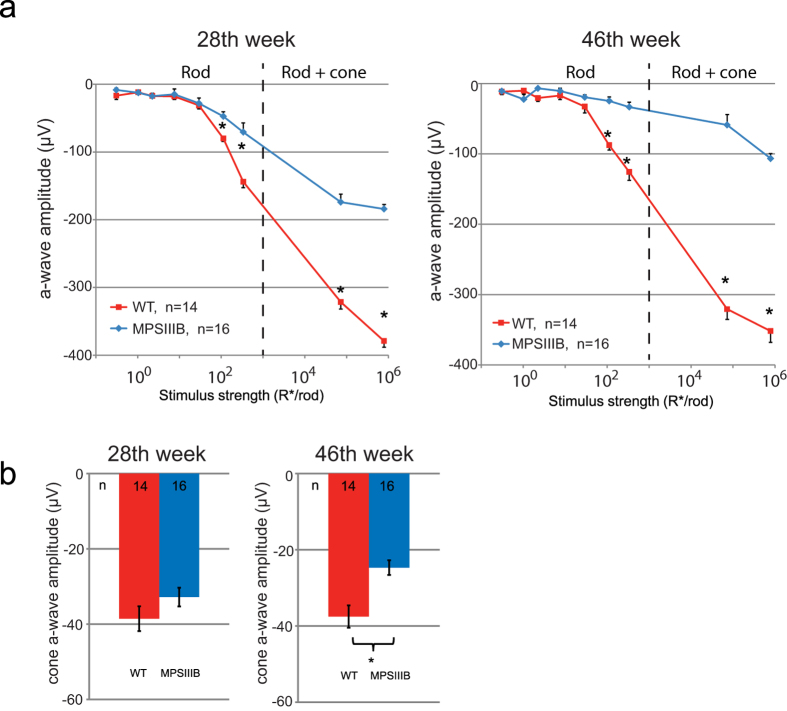
(**a**) Stimulus-response plots showing amplitudes of the dark-adapted ERG a-wave recorded from the WT and MPS IIIB mice at the 28^th^ and the 46^th^ week. The dotted lines denote the approximate threshold for cone photoreceptors based on reported data from suction-electrode recording of outer segments^67^,^68^. (**b**) Amplitudes of the cone a-wave. Data points and error bars represent mean values and standard errors, respectively. *Statistically significant difference between groups, p < 0.05, unpaired t-test.

ERG analysis of MPS IIIB mice at 28 and 46 weeks of age showed decreased a-wave amplitudes at both time points compared to age-matched WT mice (Figs 2a and 3a). The differences between groups were significant at the 4 highest stimulus levels (Fig. 3a), indicating that rod function was adversely affected in the MPS IIIB mice. Furthermore, a-wave amplitudes in the MPS IIIB group decreased with time, while the same parameters in the WT group remained relatively stable over time. A-wave responses from the two brightest stimuli were fitted using a formula devised by Cideciyan and Jacobson^41^ to determine the maximum response amplitude (R_max_) for each group (detailed method is described in our previous paper^42^). As shown in Table 1, there were significant differences in the a-wave R_max_ values between the two groups recorded at both time points. MPS IIIB mice had mean R_max_ values reduced by 60.5% and 86.2% in the 28^th^ and 46^th^ weeks, respectively.

Isolated cone a-waves measured using the paired flash method revealed cone dysfunction at a later time point (Fig. 3b). The difference in amplitudes of the cone a-wave between groups was significant in the 46^th^ week (WT: −37.5 ± 2.9 μV vs. MPS IIIB: −24.7 ± 1.9 μV, unpaired t-test, two-tailed p = 0.0008) but was not significant in the 28^th^ week. The finding that rod a-wave but not cone a-wave was suppressed in the 28^th^ week suggests that rods become dysfunctional earlier than the cones in this mouse model.

#### Function of the depolarizing bipolar cells─b-wave

**Figure 4:**
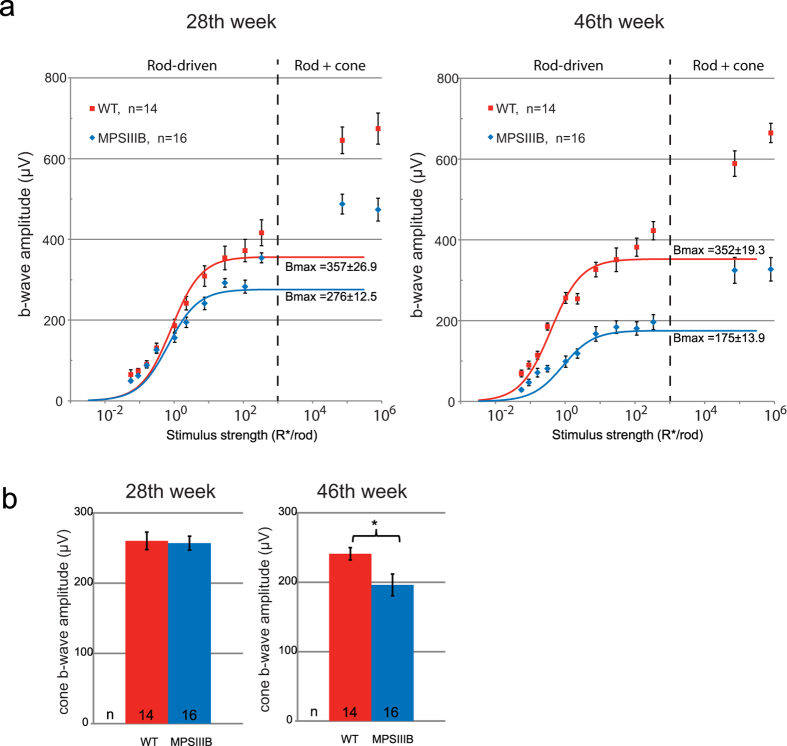
(**a**) Stimulus-response plots showing amplitudes of the dark-adapted ERG b-wave recorded from the WT and MPS IIIB mice at the 28^th^ and the 46^th^ week. The dotted lines denote the approximate threshold for cone photoreceptors. Data points within the rod operative range are fitted with sigmoidal curves using the Naka-Ruston equation. (**b**) Amplitudes of the cone b-wave. Data points and error bars represent mean values and standard errors, respectively. *Statistically significant difference between groups, p < 0.05, Welch t-test.

Decline of b-wave amplitudes in MPS IIIB mice shared a similar time course with that of the a-wave. Figure 4a shows the stimulus-response curve for the ERG b-wave in the 28^th^ and the 46^th^ weeks, fitted with the Naka-Rushton equation to determine the rod-driven b-wave maximum response amplitude (B_max_). In the 28^th^ week, MPS IIIB mice had a mean B_max_ value 22.7% lower than that of the WT mice (Welch t-test, two tailed p = 0.0142). This difference increased to 50.3% in the 46^th^ week (unpaired t-test, two tailed p < 0.0001). Using a paired-flash protocol, we found that the isolated cone b-wave in the MPS IIIB mice was not affected in the 28^th^ week but was significantly reduced in the 46^th^ week (WT: 241.0 ± 8.8 μV vs. MPS IIIB: 196.2 ± 15.6 μV, Welch’s t-test, p < 0.0053). Similar to the case of a-wave, a dysfunction in the b-wave was first detected in the rod pathway in the 28^th^ week. Function of the cone pathway appears to be preserved during the early phase of the disorder until a time point between 28–46 weeks.

#### ERG b- to a-wave ratio

The above ERG data demonstrate progressive decline of amplitudes in both the photoreceptor-generated a-wave and the bipolar cell-generated b-wave. We sought to determine whether the b-wave was directly affected by this disorder or was secondarily affected due to decreased photoreceptor input. An indirect approach to answering the above question is to evaluate the b-wave/a-wave ratios. Compared to the WT, the MPS IIIB mice had generally higher B/A ratios (Table 2). The difference was most obvious in the rod operative range, where the B/A ratio in the MPS IIIB mice was 46.2% higher in the 28^th^ week and increased to 73.5% in the 46^th^ week. The difference was smaller in the isolated cone-driven response, in which the B/A ratio in the MPS IIIB mice was 16.1% and 23.8% higher in the 28^th^ and 46^th^ week respectively. The data showed that, while both a-wave and b-wave were reduced in the MPS IIIB mice, the a-wave was suppressed by a higher percentage, leading to an increased B/A ratio in these mice. This indirect evidence supports the hypothesis that the primary site of neuronal dysfunction is represented by photoreceptors, particularly rods, although a loss of bipolar cell function is not ruled out.

### Retinal Immunohistochemistry

#### Cell profiling–vertical section

**Figure 5:**
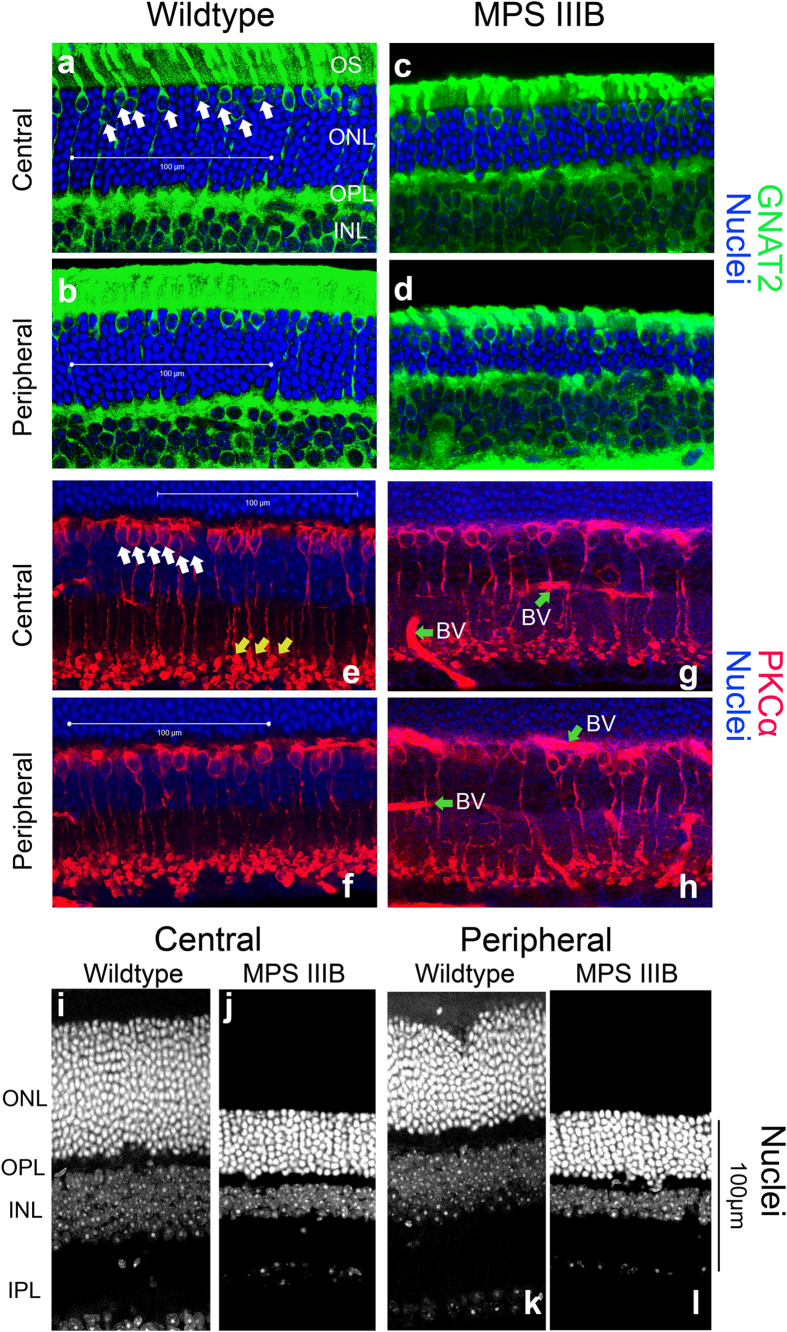
Representative confocal micrographs of retina sections taken from central and peripheral regions of WT (A,B,E,F) and MPSIIIB (C,D,G,H) mice at the 46^th^ week processed for TO-PRO3 (blue) and GNAT2 (cones; green), or TO-PRO3 (blue) and PKCα (rod bipolar cells; red). White scale bar: 100 μm. (**a–d**) TO-PRO3 labelled all photoreceptor somas in the outer nuclear layer (ONL) without differentiating rods and cones. The MPS IIIB samples have prominently reduced ONL thickness compared to the WT retina in both central and peripheral regions. Somas co-labeled by GNAT2 antibody and TO-PRO3 in (**a**–**d**) were cone somas (Some of those cells are identified by arrows in panel (**a**) as examples). There was no obvious change in the number of cone somas in the MPS IIIB retina. The green channel was enhanced to help visualizing the cones, leading to some non-specific reactivity in the inner nuclear layer (INL). (**e–h**) Rod bipolar cells were immuno-reactive to antibody for PKCα. In panel E, white arrows indicate the soma and yellow arrows indicate axonal terminals of rod bipolar cells as examples. Compared to samples from WT mice, MPSIIIB samples showed a general loss of rod bipolar cells in both regions. Green arrows denote non-specific labeling of blood vessels (BV) in G and H. Representative confocal micrographs comparing showing only nuclear staining (TO-PRO3) of WT and MPSIIIB mice at the 46^th^ week (**i–l**).

**Figure 6:**
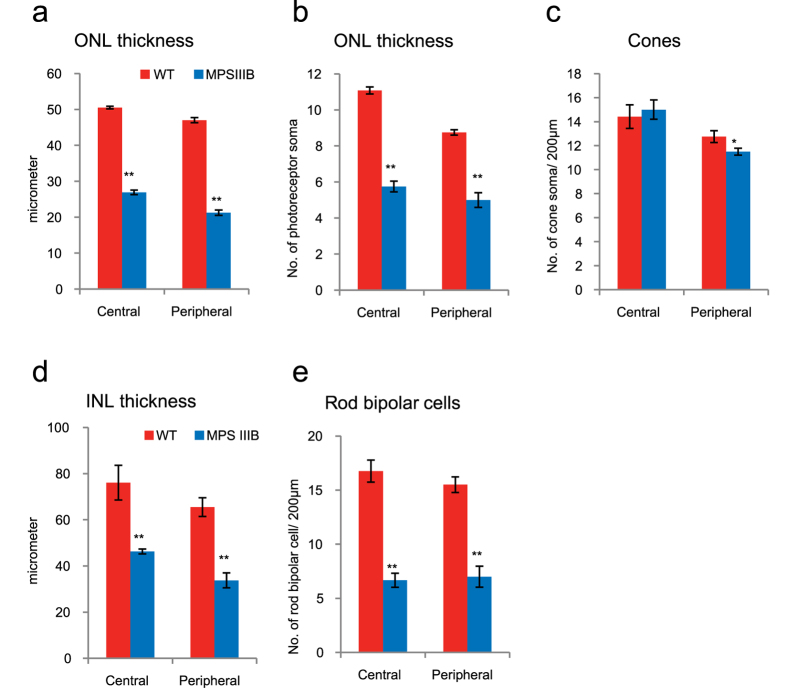
Bar charts comparing 46-week-old WT and MPS IIIB mice in terms of their retinal outer nuclear layer (ONL) thickness, number of cone soma, inner nuclear layer (INL) thickness and number of rod bipolar cells. Data points and errors bars represent group means and standard errors, respectively. Statistics performed was 2-tailed unpaired t-test, with Welch correction where appropriate. Asterisk: p < 0.05; double asterisks: p < 0.001. (**a,b**) The MPS IIIB mice showed a highly significant thinning of the ONL, due to a significant loss of photoreceptor somas. Changes in the central and peripheral regions appeared to be concomitant. (**c**) There was only a minor reduction in the number of cone somas in the peripheral retina of MPS IIIB mice. Together the data suggested that there was a major loss in the rod population in the MPS IIIB mice. (**d–e**) There was also a significant general reduction in thickness of the inner nuclear layer in the MPS IIIB mice which may be partially caused by a loss in the rod bipolar cells population.

Four mice from each genotype were sacrificed after ERG had been recorded in the 46^th^ week. One eye from each animal was microtome-sectioned and processed for immunohistochemistry analysis. Three retinal sections from each eye were imaged and had their cells counted and dimensions measured, resulting in a sample size equal to 12 for each group. As shown in the representative micrographs (Fig. 5), there were degenerative changes in both the outer nuclear layer (ONL) and inner nuclear layer (INL). In particular, the mean ONL thicknesses of MPS IIIB mice was 46.7% and 54.8% thinner than that of WT mice in the central and peripheral regions, respectively (Fig. 6a–c). In terms of photoreceptor somas in the ONL, the MPS IIIB mice had lost 48.1% and 42.6% in the central and peripheral regions, respectively. Since the TO-PRO3 dye stained both rod and cone somas, we utilized a cone-specific anti-GNAT2 antibody to determine if there was a differential loss in cones versus rods. The data showed that there was no significant difference between WT and MPS IIIB mice in the number of cone somas present in the ONL in the central region, and that MPS IIIB mice had 9.8% fewer cone somas in the peripheral region. Together, the large reduction in the number of photoreceptors and the minor reduction in the number of cones suggest that the thinning of ONL was primarily caused by a loss of rods in the MPS IIIB mice. We also found that the INL thickness of the MPS IIIB mice was 39.2% and 48.4% thinner compared to WT mice in the central and peripheral regions, respectively (Fig. 6d,e). In an attempt to identify the retinal cell type underlying the loss in the INL, we used antibodies for protein kinase C alpha (PKCα), a marker for rod bipolar cells (BC_R_s). Our data showed that the BC_R_ population had decreased by 60.2% and 54.8%, respectively, in the central and peripheral regions.

#### Inflammatory cell makers and neovascularization–flat-mount

**Figure 7:**
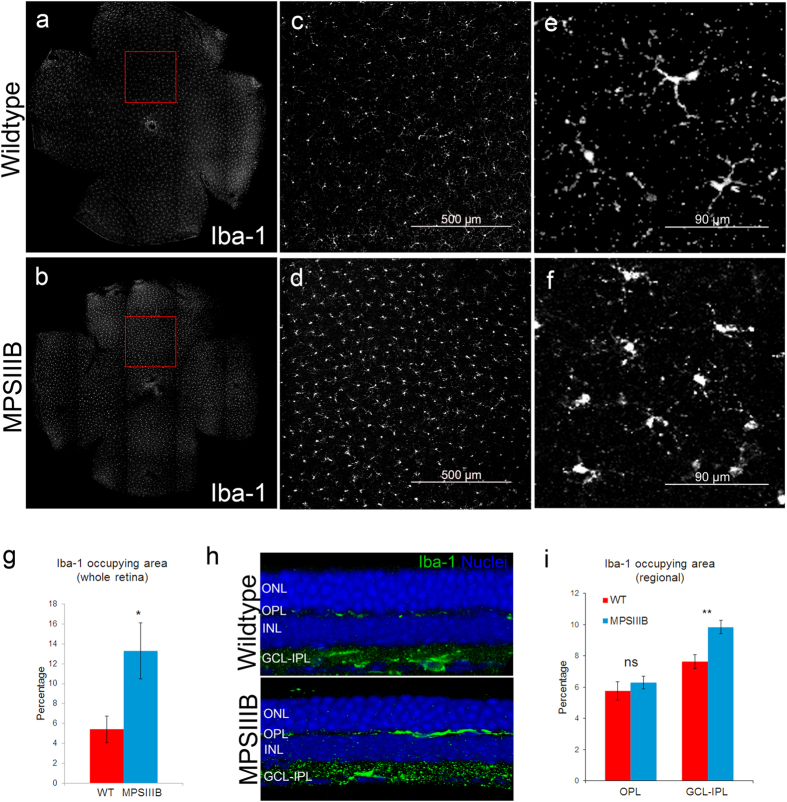
Representative tile-scan micrographs of whole-mount retina processed for activated microglia (Iba-1) from (a) WT and (b) MPS IIIB mice at the 46^th^ week. Panels (**c**,**d**) are medium magnification views of the selected regions in (**a**,**b**), respectively. Scale bar: 500 μm. The density of activated microglial cells is higher in samples from MPS IIIB mice. High magnification views (**e**,**f**) showed that microglia displays a more ramified appearance in the WT retina, and a less ramified amoeboid shape in the MPS IIIB retina. (**g**) Bar charts comparing the percentage retinal area occupied by Iba-1-positive cells. Data points and errors bars represent group means and standard errors, respectively. (unpaired t-test with Welch correction. Asterisk: p < 0.05). Both cell density and occupied area were found to be significantly higher in the MPS IIIB sample. (**h**) Micrographs of MPSIII retinal sections showing the stratification of Iba-1 positive cells, which were present in the layers of OPL and GCL-IPL. (**i**) Bar chart showing percentage area occupied by Iba-1-positive cells were higher specifically in the GCL-IPL layer. (unpaired t-test. Asterisk: p < 0.01).

Four WT and three MPS IIIB mice from a different cohort were sacrificed in the 46^th^ week. Whole-mount retinas from both eyes were processed for immunohistochemistry and cell counting, giving sample sizes of 8 and 6 for the WT and MPS IIIB groups, respectively. There were marked differences between MPS IIIB and WT mice in Iba1-positive cells morphologically (Fig. 7a–f). After examined the tile-scanned whole retina image, we found that the retinal area occupied by the Iba-1-positive cells was much higher in the MPS IIIB mice (Fig. 7g). Under imageJ particle analysis, the cells occupied 13.3% and 5.4% of the retinal area in MPS IIIB and WT mice, respectively (Welch-t test, two-tailed p = 0.026). Further analysis of sectioned MPS IIIB retina revealed that Iba-1 positive cells are located at the OPL and the ganglion cell layer-inner plexiform layer (GCL-IPL). Therefore the relative change of Iba-1 positive cells in the two layers were further analyzed separately, by processing z-stack images on representative regions of the flat-mounted retinas. The results showed that the percentage area occupied by Iba-1 positive cells were specifically higher at the GCL-IPL (Fig. 7h,i).

**Figure 8:**
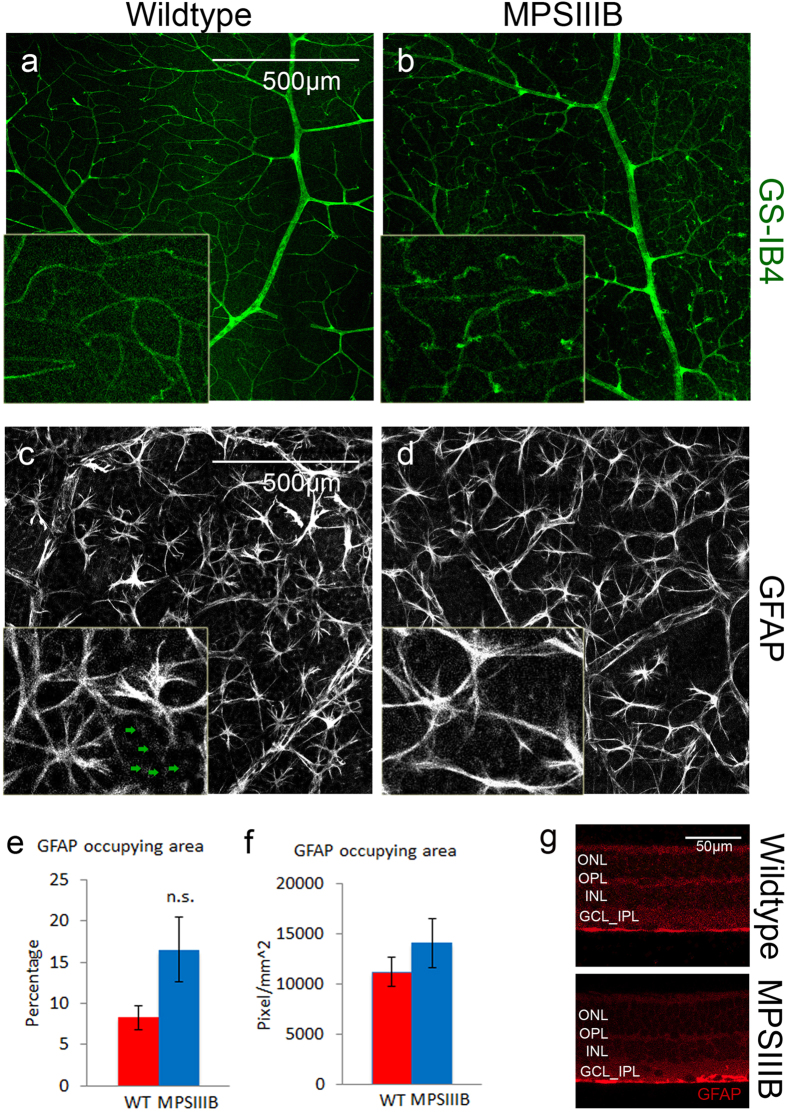
(**a**,**b**) GS-IB4 labeled retinal vasculature in whole mount WT and MPSIIIB retina at the 46^th^ week. Scale bar: 500 μm. Insets showing a magnified view of the neo-vascular tufts. Note the presence of neo-vascular tufts throughout the vasculature in MPSIIIB retina but not in the WT retina. (**c,d**) GFAP labeled astrocyte (bright star-shaped cells), and end-feet of Müller cells (appeared as dim punctate staining; denote by green arrows in (**c**)). Morphology of astrocytes appeared similar in WT and MPSIIIB retina. There was no observable change in GFAP reactivity of Müller cells in MPSIIIB retina. (**e**,**f**) No difference was found in area occupied by GFAP positive cells. (**g**) Retinal sections showing GFAP reactivity was localized in the GCL layer of MPSIIIB retina

Retinal vasculature labelled with GS-IB4 identified the presence of neo-vascular tufts in MPSIIIB retina but not in WT retina (Fig. 8a,b).

There were no significant differences between WT and MPSIIIB retina in GFAP reactivity on reactive astrocytes and Müller cells (Fig. 8e–g). The morphology of astrocytes in MPSIIIB retina was similar to those in WT. No obvious loss of secondary cell processes was observed (Fig. 8c,d). Also, GFAP-reactive astrocyte/Müller cell processes seemed to be limited in GCL layer in both WT and MPSIIIB (Fig. 8g).

## Discussion

In the present study, we have characterized the functional and morphological changes that occur in the retina of the Sanfilippo syndrome Type B (MPS IIIB) mouse model. Our findings are in agreement with, and significantly extend, a previous screening study of this model^28^. The decline of the ERG rod a-wave by 60% and 86% in the 28^th^ and the 46^th^ postnatal weeks, respectively, were most remarkable. In the 46^th^ week, 43-48% of photoreceptor somas were lost (rods to a greater extent than cones). Furthermore, we found that the cone pathway began to show signs of dysfunction late in the disease course. Both the ERG cone a- and b-waves were significantly smaller in the KOs. A roughly 10% reduction of cones photoreceptors was found in the peripheral retina of the KOs. This defect in the cone pathway was not detected in the previous study of this mouse model, probably because cone degeneration is not evident until a late stage of the disease (>40 postnatal weeks). It was also surprising to find that rod bipolar cells (BC_R_s), the secondary neurons postsynaptic to the rods, had also degenerated and lost more than 50% of cells by the 46^th^ week. Analysis of the B/A wave ratio indicated that the a-wave declined to a greater extent than the b-wave, thus providing indirect support that the primary site of degeneration was the rods rather than the rod bipolar cells. Another novel finding is that the density of activated microglia in MPS IIIB mice was approximately triple compared with that in WT mice at the 46^th^ week time point, indicating that retinal inflammation might be involved in the degeneration. In addition, neo-vascularization was found to be present in the MPSIII retina as well.

The most prominent retinal change in the MPS IIIB mouse model was the dysfunction and degeneration of photoreceptors. While the exact mechanism remains elusive, it is likely to be linked to the lysosomal inclusion and accumulation of HS at the retinal pigment epithelium (RPE) as a result of enzymatic deficiency. The RPE is known to be crucial for sustaining the photoreceptors by serving a number of functions including the regulation of ion and metabolite transport^43^, retinoid transport^44^, and the phagocytosis of outer segments^45^. GAGs including HS are synthesized and secreted by the RPE under physiological conditions^46^,^47^. In MPS III patients, lysosome inclusion or lysosomal storage vacuoles are present in large numbers in the RPE^23^,^24^. Similar melanosome-like structures were also found in the RPE of 30-week-old MPS IIIB mice^28^. Though there is no specific experimental evidence for the role of RPE in photoreceptor degeneration in MPS III, previous studies using cultured MPS VII RPE cells have shown that the accumulation of GAGs in RPE cells was associated with the deficiency of the corresponding enzyme (β-glucuronidase) for their degradation^48^, and that genetically restoring the enzymatic activity of RPE cells using retrovirus reduced accumulation of GAGs *in vitro*^48^. Furthermore, supplementing the missing β-glucuronidase activity in RPE by transferring the correct gene to retinal cells through intravitreally injected adeno-associated virus improved the ERG *in vivo*^49^.

A noteworthy feature of the MPS IIIB mouse model is that rod degeneration was evident much earlier than that of the cones. This finding is consistent with the typical clinical signs and symptoms of MPS III. These patients often report their first visual symptom as night blindness, while some of them have near normal visual acuity under photopic conditions^25^. Their rod-driven ERG is also reduced to a greater extent than the cone-driven ERG^50^. A possible explanation for the differential impact of the disease on rods versus cones could be that rods are more sensitive to the disrupted supportive function of RPE induced by HS. It is also possible that a bystander effect from rod degeneration may contribute to the degeneration of cones. Numerous studies have shown that degeneration of rods induced by rod-specific genetic defects may spread to neighboring cones through an unclear mechanism^51^,^52^.

The above rod-cone sequence of loss in MPS III is common among patients with MPS I and MPS II as well^25^, but different trends were found in a MPS VII animal model^53^,^54^. One possible explanation for this discrepancy could be difference in the composition of the primary accumulated GAGs in the different MPS types. MPS VII, or Sly syndrome, is known to have chondroitin sulfate as an undegraded GAG in addition to HS^53^. It is yet to be determined whether chondroitin sulfate is particularly deleterious for cones.

Our immunohistochemistry finding that rod bipolar cells (BC_R_s) had undergone significant degeneration was unexpected because it is commonly believed that MPS III-associated retinopathy originates at the RPE^48^,^49^,^53^. However, this finding can be interpreted by taking into consideration that BC_R_s degeneration also takes place in aged patients of non-MPS retinitis pigmentosa (RP), as well as in mouse strains that have retinal degeneration caused by sole mutations in rods^55^. A cell loss of 20–60% in the inner nuclear layer has been observed in RP patients, whereas greater loss was found in extramacular region^56^,^57^. Reduced complexity of BC_R_s dendrites^58^, and a 20% loss of BC_R_s in period of 2 months were also observed in mouse RP models^55^. Thus, BC_R_ degeneration appeared to be independent of the primary insult deriving from the underlying genetic defect and to rather be a result of the secondary effects of photoreceptor death. One hypothesis is that BC_R_ requires a normal glutamatergic synaptic input or some unknown factors secreted from the rods to maintain its validity, and the deprivation of those factors causes the degeneration.

An alternative hypothesis specific for the BC_R_ degeneration in MPSIII is that the activation of microglia and the subsequent inflammatory response exacerbate the death of BC_R_s. This notion is supported by our immunohistochemistry data, which showed obvious activation of retinal microglia. Additional support to this notion comes from the aberrant lysosome storage found in non-neuronal cells in the inner nuclear layer of MPS IIIB mouse retina^28^. Indeed, microglial over-activation was suggested to be involved in the pathogenesis of MPS III^8^,^9^, as well as in some other disorders of the central nervous system such as Alzheimer’s disease and Parkinson’s disease^59^,^60^,^61^. In the eye, microglial activation is associated with, and sometimes precedes, photoreceptor apoptosis and retinal degeneration^62^,^63^. Neo-vascularization previously reported in the heart valve and cartilage of MPSVII dogs^64^,^65^ was also found on MPSIIIB retina. Its link with microglia activation is yet to be determined. In contrast, morphological changes in astrocytes and increased GFAP reactivity in Müller cells, previously reported to accompany microglial activation during retinal injuries (such as ocular hypertension and light damage^39^,^66^), was not observed in our MPSIIIB model.

In the present study, we have performed detailed analyses of the retinal electrophysiological and morphological changes in a mouse model of MPS IIIB. The slow progression of retinal degeneration from normal to mild dysfunction and eventually massive cell loss provides a large time window and numerous measurable parameters for experimental manipulations. We have also shown that the ERG is a reliable, non-invasive and quantitative test to longitudinally monitor the progression of the retinopathy, and it has the potential to determine the effectiveness of future ocular/systemic therapy. Finally, the current interpretation of MPS IIIB retinopathy is based on a model in which rod degeneration following accumulation of GAGs at the RPE drives pathogenesis and degeneration. However, our findings that degeneration of rod bipolar cells and cones photoreceptor also takes place indicate that the RPE may not be the only primary site of the disease and imply that additional measure (e.g. anti-inflammation, neurotropic factors) may be needed to protect vision in MPSIII patients when treatments are being developed in the future. The fact that retinal microglia activation is involved in the degeneration implies that modulating retinal inflammation may be beneficial to delay the retinal degeneration, which warrants further investigation.

## Additional Information

**How to cite this article**: Tse, D. Y. *et al*. Electrophysiological and Histological Characterization of Rod-Cone Retinal Degeneration and Microglia Activation in a Mouse Model of Mucopolysaccharidosis Type IIIB. *Sci. Rep*. **5**, 17143; doi: 10.1038/srep17143 (2015).

## Figures and Tables

**Table 1:** ERG scotopic a-wave analysis.

Age (wk)	Group	R_max_ ± SEM (μV)	R_max_ (%) w.r.t. WT	n	P
28	WT	316.8 ± 8.6	100	13	<0.0001
	MPS IIIB	125.2 ± 6.0	39.5	16	Unpaired t-test
46	WT	323.9 ± 8.2	100	14	<0.0001
	MPS IIIB	44.8 ± 3.9	13.8	16	Welch’s t-test

A-wave responses from the two brightest stimuli were fitted using a formula devised by Cideciyan and Jacobson^41^ to determine the maximum response amplitude (R_max_). The percentage R_max_ values are with reference to the R_max_ of WT.

**Table 2:** Ratios of b-wave/a-wave. Values for rod-driven ERG were calculated from scotopic ERG data measured using stimulus strength of 112.2 isomerizations/rod, which was below the threshold for cones.

Rod/cone Origin	Group	b/a(28^th^ week)	b/a (%) w.r.t. WT	b/a (46^th^ week)	b/a (%) w.r.t. WT
**Rod**	WT	4.94	100	4.41	100
	MPS IIIB	7.22	146.2	7.65	173.5
**Cone**	WT	6.75	100	6.42	100
	MPS IIIB	7.83	116.1	7.96	123.8

Values for cone-driven ERG were calculated from paired-flash data, in which rod activity was effectively suppressed.

## References

[b1] Walkley S. U. Pathogenic cascades in lysosomal disease-Why so complex? Journal of inherited metabolic disease 32, 181–189, doi: 10.1007/s10545-008-1040-5 (2009). 19130290 PMC2682782

[b2] Platt F. M. W. & S. U. In Lysosomal Disorders of the Brain (ed Walkley Platt F. M. , S.U.) Ch. 2, 32–49 (Oxford University Press, 2004).

[b3] Sardiello M. . A gene network regulating lysosomal biogenesis and function. Science 325, 473–477, doi: 10.1126/science.1174447 (2009). 19556463

[b4] Sardiello M. & Ballabio A. Lysosomal enhancement: a CLEAR answer to cellular degradative needs. Cell cycle 8, 4021–4022 (2009). 19949301 10.4161/cc.8.24.10263

[b5] Capila I. & Linhardt R. J. Heparin-protein interactions. Angewandte Chemie 41, 391–412 (2002). 12491369 10.1002/1521-3773(20020201)41:3<390::aid-anie390>3.0.co;2-b

[b6] Jones M. Z. . Human mucopolysaccharidosis IIID: clinical, biochemical, morphological and immunohistochemical characteristics. Journal of neuropathology and experimental neurology 56, 1158–1167 (1997). 9329460

[b7] Walkley S. U., Siegel D. A. & Dobrenis K. GM2 ganglioside and pyramidal neuron dendritogenesis. Neurochemical research 20, 1287–1299 (1995). 8786814 10.1007/BF00992503

[b8] Ohmi K. . Activated microglia in cortex of mouse models of mucopolysaccharidoses I and IIIB. Proc Natl Acad Sci USA 100, 1902–1907, doi: 10.1073/pnas.252784899 (2003). 12576554 PMC149931

[b9] Villani G. R. . Cytokines, neurotrophins, and oxidative stress in brain disease from mucopolysaccharidosis IIIB. J Neurosci Res 85, 612–622, doi: 10.1002/jnr.21134 (2007). 17139681

[b10] Valstar M. J., Ruijter G. J., van Diggelen O. P., Poorthuis B. J. & Wijburg F. A. Sanfilippo syndrome: a mini-review. Journal of inherited metabolic disease 31, 240–252, doi: 10.1007/s10545-008-0838-5 (2008). 18392742

[b11] Baehner F. . Cumulative incidence rates of the mucopolysaccharidoses in Germany. Journal of inherited metabolic disease 28, 1011–1017, doi: 10.1007/s10545-005-0112-z (2005). 16435194

[b12] Meikle P. J., Hopwood J. J., Clague A. E. & Carey W. F. Prevalence of lysosomal storage disorders. JAMA: the journal of the American Medical Association 281, 249–254 (1999). 10.1001/jama.281.3.249 9918480

[b13] Poorthuis B. J. . The frequency of lysosomal storage diseases in The Netherlands. Hum Genet 105, 151–156 (1999). 10480370 10.1007/s004399900075

[b14] Yogalingam G. & Hopwood J. J. Molecular genetics of mucopolysaccharidosis type IIIA and IIIB: Diagnostic, clinical, and biological implications. Human mutation 18, 264–281, doi: 10.1002/humu.1189 (2001). 11668611

[b15] Weber B. . Sanfilippo type B syndrome (mucopolysaccharidosis III B): allelic heterogeneity corresponds to the wide spectrum of clinical phenotypes. European journal of human genetics: EJHG 7, 34–44, doi: 10.1038/sj.ejhg.5200242 (1999). 10094189

[b16] Martin J. J., Ceuterick C., Van Dessel G., Lagrou A. & Dierick W. Two cases of mucopolysaccharidosis type III (Sanfilippo). An anatomopathological study. Acta neuropathologica 46, 185–190 (1979). 223363 10.1007/BF00690842

[b17] van de Kamp J. J., Niermeijer M. F., von Figura K. & Giesberts M. A. Genetic heterogeneity and clinical variability in the Sanfilippo syndrome (types A, B, and C). Clinical genetics 20, 152–160 (1981). 6796310 10.1111/j.1399-0004.1981.tb01821.x

[b18] Meyer A. . Scoring evaluation of the natural course of mucopolysaccharidosis type IIIA (Sanfilippo syndrome type A). Pediatrics 120, e1255–1261, doi: 10.1542/peds.2007-0282 (2007). 17938166

[b19] Ruijter G. J. . Clinical and genetic spectrum of Sanfilippo type C (MPS IIIC) disease in The Netherlands. Molecular genetics and metabolism 93, 104–111, doi: 10.1016/j.ymgme.2007.09.011 (2008). 18024218

[b20] Ashworth J. L., Biswas S., Wraith E. & Lloyd I. C. Mucopolysaccharidoses and the eye. Survey of ophthalmology 51, 1–17, doi: 10.1016/j.survophthal.2005.11.007 (2006). 16414358

[b21] Alroy J., Haskins M. & Birk D. E. Altered corneal stromal matrix organization is associated with mucopolysaccharidosis I, III and VI. Exp Eye Res 68, 523–530, doi: 10.1006/exer.1998.0622 (1999). 10328965

[b22] Berger-Plantinga E. G., Vanneste J. A., Groener J. E. & van Schooneveld M. J. Adult-onset dementia and retinitis pigmentosa due to mucopolysaccharidosis III-C in two sisters. Journal of neurology 251, 479–481, doi: 10.1007/s00415-004-0368-5 (2004). 15083297

[b23] Lavery M. A., Green W. R., Jabs E. W., Luckenbach M. W. & Cox J. L. Ocular histopathology and ultrastructure of Sanfilippo’s syndrome, type III-B. Arch Ophthalmol 101, 1263–1274 (1983). 6309125 10.1001/archopht.1983.01040020265021

[b24] Del Monte M. A., Maumenee I. H., Green W. R. & Kenyon K. R. Histopathology of Sanfilippo’s syndrome. Arch Ophthalmol 101, 1255–1262 (1983). 6411049 10.1001/archopht.1983.01040020257020

[b25] Ashworth J. L., Biswas S., Wraith E. & Lloyd I. C. The ocular features of the mucopolysaccharidoses. Eye (Lond) 20, 553–563, doi: 10.1038/sj.eye.6701921 (2006). 15905869

[b26] Li H. H. . Mouse model of Sanfilippo syndrome type B produced by targeted disruption of the gene encoding alpha-N-acetylglucosaminidase. Proc Natl Acad Sci USA 96, 14505–14510 (1999). 10588735 10.1073/pnas.96.25.14505 PMC24466

[b27] Li H. H., Zhao H. Z., Neufeld E. F., Cai Y. & Gomez-Pinilla F. Attenuated plasticity in neurons and astrocytes in the mouse model of Sanfilippo syndrome type B. J Neurosci Res 69, 30–38, doi: 10.1002/jnr.10278 (2002). 12111813

[b28] Heldermon C. D. . Development of sensory, motor and behavioral deficits in the murine model of Sanfilippo syndrome type B. PLoS One 2, e772, doi: 10.1371/journal.pone.0000772 (2007). 17712420 PMC1945015

[b29] Dowling J. E. The retina: an approachable part of the brain. 2nd edition edn, (Belknap Press of Harvard University Press, 2012).

[b30] Mauri V., Lotfi P., Segatori L. & Sardiello M. A rapid and sensitive method for measuring N-acetylglucosaminidase activity in cultured cells. PLoS One 8, e68060, doi: 10.1371/journal.pone.0068060 (2013). 23840811 PMC3695942

[b31] Saszik S. M., Robson J. G. & Frishman L. J. The scotopic threshold response of the dark-adapted electroretinogram of the mouse. J Physiol 543, 899–916 (2002). 12231647 10.1113/jphysiol.2002.019703 PMC2290546

[b32] Abd-El-Barr M. M. . Genetic dissection of rod and cone pathways in the dark-adapted mouse retina. J Neurophysiol 102, 1945–1955, doi: 10.1152/jn.00142.2009 (2009). 19587322 PMC2746771

[b33] Pennesi M. E., Howes K. A., Baehr W. & Wu S. M. Guanylate cyclase-activating protein (GCAP) 1 rescues cone recovery kinetics in GCAP1/GCAP2 knockout mice. Proc Natl Acad Sci USA 100, 6783–6788, doi: 10.1073/pnas.1130102100 (2003). 12732716 PMC164524

[b34] Vekslin S. & Ben-Yosef T. Spatiotemporal expression pattern of ceramide kinase-like in the mouse retina. Mol Vis 16, 2539–2549 (2010). 21151604 PMC3000240

[b35] Zhang J., Yang Z. & Wu S. M. Development of cholinergic amacrine cells is visual activity-dependent in the postnatal mouse retina. J Comp Neurol 484, 331–343, doi: 10.1002/cne.20470 (2005). 15739235

[b36] Chang B. . Cone photoreceptor function loss-3, a novel mouse model of achromatopsia due to a mutation in Gnat2. Invest Ophthalmol Vis Sci 47, 5017–5021, doi: 10.1167/iovs.05-1468 (2006). 17065522

[b37] Kostic C. . Gene therapy regenerates protein expression in cone photoreceptors in Rpe65(R91W/R91W) mice. PLoS ONE 6, e16588, doi: 10.1371/journal.pone.0016588 (2011). 21304899 PMC3033393

[b38] Ma W., Zhao L., Fontainhas A. M., Fariss R. N. & Wong W. T. Microglia in the mouse retina alter the structure and function of retinal pigmented epithelial cells: a potential cellular interaction relevant to AMD. PLoS One 4, e7945, doi: 10.1371/journal.pone.0007945 (2009). 19936204 PMC2775955

[b39] Gallego B. I. . IOP induces upregulation of GFAP and MHC-II and microglia reactivity in mice retina contralateral to experimental glaucoma. Journal of neuroinflammation 9, 92, doi: 10.1186/1742-2094-9-92 (2012). 22583833 PMC3410794

[b40] Tual-Chalot S., Allinson K. R., Fruttiger M. & Arthur H. M. Whole mount immunofluorescent staining of the neonatal mouse retina to investigate angiogenesis *in vivo*. J Vis Exp, e50546, doi: 10.3791/50546 (2013). 23892721 PMC3732076

[b41] Cideciyan A. V. & Jacobson S. G. An alternative phototransduction model for human rod and cone ERG a-waves: normal parameters and variation with age. Vision research 36, 2609–2621 (1996). 8917821 10.1016/0042-6989(95)00327-4

[b42] Simons D. L., Boye S. L., Hauswirth W. W. & Wu S. M. Gene therapy prevents photoreceptor death and preserves retinal function in a Bardet-Biedl syndrome mouse model. Proceedings of the National Academy of Sciences of the United States of America 108, 6276–6281, doi: 10.1073/pnas.1019222108 (2011). 21444805 PMC3076852

[b43] Steinberg R. H. & Miller S. S. In The Retinal Pigment Epithelium (eds Zinn K. & Marmor M. ) 205–225 (Harvard University Press, 1979).

[b44] 44Bok D. & Heller J. Transport of retinol from the blood to the retina: an autoradiographic study of the pigment epithelial cell surface receptor for plasma retinol-binding protein. Exp Eye Res 22, 395–402 (1976). 945179 10.1016/0014-4835(76)90177-9

[b45] Young R. W. & Bok D. Participation of the retinal pigment epithelium in the rod outer segment renewal process. The Journal of cell biology 42, 392–403 (1969). 5792328 10.1083/jcb.42.2.392 PMC2107669

[b46] Robey P. G. & Newsome D. A. Biosynthesis of proteoglycans present in primate Bruch’s membrane. Invest Ophthalmol Vis Sci 24, 898–905 (1983). 6862794

[b47] Turksen K., Aubin J. E., Sodek J. & Kalnins V. I. Localization of laminin, type IV collagen, fibronectin, and heparan sulfate proteoglycan in chick retinal pigment epithelium basement membrane during embryonic development. The journal of histochemistry and cytochemistry: official journal of the Histochemistry Society 33, 665–671 (1985). 3159787 10.1177/33.7.3159787

[b48] Stramm L. E. . Beta-glucuronidase mediated pathway essential for retinal pigment epithelial degradation of glycosaminoglycans. Disease expression and *in vitro* disease correction using retroviral mediated cDNA transfer. Exp Eye Res 50, 521–532 (1990). 2164946 10.1016/0014-4835(90)90041-r

[b49] Hennig A. K. . AAV-mediated intravitreal gene therapy reduces lysosomal storage in the retinal pigmented epithelium and improves retinal function in adult MPS VII mice. Molecular therapy: the journal of the American Society of Gene Therapy 10, 106–116, doi: 10.1016/j.ymthe.2004.03.018 (2004). 15233947

[b50] Caruso R. C. . Electroretinographic findings in the mucopolysaccharidoses. Ophthalmology 93, 1612–1616 (1986). 3101020 10.1016/s0161-6420(86)33537-1

[b51] Ripps H. Cell death in retinitis pigmentosa: gap junctions and the ‘bystander’ effect. Exp Eye Res 74, 327–336, doi: 10.1006/exer.2002.1155 (2002). 12014914

[b52] Kranz K., Paquet-Durand F., Weiler R., Janssen-Bienhold U. & Dedek K. Testing for a gap junction-mediated bystander effect in retinitis pigmentosa: secondary cone death is not altered by deletion of connexin36 from cones. PLoS One 8, e57163, doi: 10.1371/journal.pone.0057163 (2013). 23468924 PMC3584123

[b53] Lazarus H. S., Sly W. S., Kyle J. W. & Hageman G. S. Photoreceptor degeneration and altered distribution of interphotoreceptor matrix proteoglycans in the mucopolysaccharidosis VII mouse. Exp Eye Res 56, 531–541, doi: 10.1006/exer.1993.1067 (1993). 8500564

[b54] Ohlemiller K. K., Vogler C. A., Roberts M., Galvin N. & Sands M. S. Retinal function is improved in a murine model of a lysosomal storage disease following bone marrow transplantation. Exp Eye Res 71, 469–481, doi: 10.1006/exer.2000.0897 (2000). 11040082

[b55] Gargini C., Terzibasi E., Mazzoni F. & Strettoi E. Retinal organization in the retinal degeneration 10 (rd10) mutant mouse: a morphological and ERG study. J Comp Neurol 500, 222–238, doi: 10.1002/cne.21144 (2007). 17111372 PMC2590657

[b56] Santos A. . Preservation of the inner retina in retinitis pigmentosa. A morphometric analysis. Arch Ophthalmol 115, 511–515 (1997). 9109761 10.1001/archopht.1997.01100150513011

[b57] Humayun M. S. . Morphometric analysis of the extramacular retina from postmortem eyes with retinitis pigmentosa. Invest Ophthalmol Vis Sci 40, 143–148 (1999). 9888437

[b58] Strettoi E. & Pignatelli V. Modifications of retinal neurons in a mouse model of retinitis pigmentosa. Proc Natl Acad Sci USA 97, 11020–11025, doi: 10.1073/pnas.190291097 (2000). 10995468 PMC27141

[b59] Maccioni R. B., Rojo L. E., Fernandez J. A. & Kuljis R. O. The role of neuroimmunomodulation in Alzheimer’s disease. Ann N Y Acad Sci 1153, 240–246, doi: 10.1111/j.1749-6632.2008.03972.x (2009). 19236346

[b60] Farooqui T. & Farooqui A. A. Lipid-mediated oxidative stress and inflammation in the pathogenesis of Parkinson’s disease. Parkinson’s disease 2011, 247467, doi: 10.4061/2011/247467 (2011). PMC3042619 21403820

[b61] Fuhrmann M. . Microglial Cx3cr1 knockout prevents neuron loss in a mouse model of Alzheimer’s disease. Nat Neurosci 13, 411–413, doi: 10.1038/nn.2511 (2010). 20305648 PMC4072212

[b62] Zeng H. Y. . Identification of sequential events and factors associated with microglial activation, migration, and cytotoxicity in retinal degeneration in rd mice. Invest Ophthalmol Vis Sci 46, 2992–2999, doi: 10.1167/iovs.05-0118 (2005). 16043876

[b63] Zeiss C. J. & Johnson E. A. Proliferation of microglia, but not photoreceptors, in the outer nuclear layer of the rd-1 mouse. Invest Ophthalmol Vis Sci 45, 971–976 (2004). 14985319 10.1167/iovs.03-0301

[b64] Xing E. M. . The effect of neonatal gene therapy on skeletal manifestations in mucopolysaccharidosis VII dogs after a decade. Molecular genetics and metabolism 109, 183–193, doi: 10.1016/j.ymgme.2013.03.013 (2013). 23628461 PMC3690974

[b65] Bigg P. W. . Pathogenesis of mitral valve disease in mucopolysaccharidosis VII dogs. Molecular genetics and metabolism 110, 319–328, doi: 10.1016/j.ymgme.2013.06.013 (2013). 23856419 PMC3800211

[b66] de Raad S., Szczesny P. J., Munz K. & Reme C. E. Light damage in the rat retina: glial fibrillary acidic protein accumulates in Muller cells in correlation with photoreceptor damage. Ophthalmic research 28, 99–107 (1996). 8792360 10.1159/000267881

[b67] Nikonov S. S., Kholodenko R., Lem J. & Pugh E. N. Jr. Physiological features of the S- and M-cone photoreceptors of wild-type mice from single-cell recordings. J Gen Physiol 127, 359–374, doi: 10.1085/jgp.200609490 (2006). 16567464 PMC2151510

[b68] Field G. D. & Rieke F. Nonlinear signal transfer from mouse rods to bipolar cells and implications for visual sensitivity. Neuron 34, 773–785 (2002). 12062023 10.1016/s0896-6273(02)00700-6

